# O Impacto Das Doenças Cardiovasculares Nas Perdas Econômicas

**DOI:** 10.36660/abc.20240175

**Published:** 2024-05-07

**Authors:** Fátima Marinho

**Affiliations:** 1 Vital Strategies Brazil Nova Iorque EUA Vital Strategies Brazil, Nova Iorque – EUA; 2 Universidade Federal de Minas Gerais Belo Horizonte MG Brasil Universidade Federal de Minas Gerais (UFMG), Belo Horizonte, MG – Brasil

**Keywords:** Doenças Cardiovasculares/mortalidade, Políticas Públicas em Saúde/economia, Análise Custo-Eficiência

As doenças cardiovasculares (DCV) são um problema de saúde pública que impacta a saúde e a economia da população. É a principal causa de morte no mundo^1^ No Brasil, as DCV têm sido a principal causa de morte, sendo a Cardiopatia Isquêmica e o acidente vascular cerebral as causas mais comuns, exceto durante os anos de pandemia (2020-2021), quando a COVID-19 foi a primeira causa de morte em homens e mulheres.^2^

Qual é o impacto no trabalho produtivo e na economia? Como prevenir as DCV e a perda de saúde da população?

As DCV impactam significativamente a produtividade do trabalho, resultando em uma perda anual de produtividade de US$ 147 bilhões nos EUA.^3^ Indivíduos com alto risco cardiovascular experimentam mais horas perdidas de trabalho e custos indiretos mais elevados em comparação com aqueles sem eventos cardiovasculares. Na Austrália, a investigação sobre a prevenção das doenças coronárias durante dez anos revelou que isso salvaria muitas vidas e quase 15 mil milhões de dólares em produto interno bruto (PIB).^4^ Na União Europeia,^5^ as DCV também tiveram um impacto significativo, com custos estimados em perdas de produtividade ascendendo a 48 mil milhões de euros (17%).

Na América do Sul, onde o impacto das DCV é particularmente pronunciado, os esforços para quantificar as consequências económicas destas doenças têm sido limitados. O estudo de Bandeira et al.,^6^ fornece uma contribuição valiosa para preencher esta lacuna, estimando os anos de vida produtiva perdidos (AVPP) e os custos económicos associados à mortalidade prematura por DCV em 2019.

É importante notar que existem algumas limitações na utilização de dados relacionados às DCV, que são cruciais para as políticas de saúde pública. Nem todos os países dispõem de dados fiáveis sobre a mortalidade por DCV. Entre 156 países, apenas 70% das mortes foram registadas e, destes, apenas 52% tiveram causas clinicamente certificadas.^7^ Isto torna necessário confiar em estimativas das causas de morte.

A pesquisa conduzida por Bandeira et al.,^6^ utiliza dados do *Global Burden of Disease Study 2019* , que é um banco de dados abrangente e amplamente utilizado. O estudo tem como objetivo estimar a carga de DCV na América do Sul. Fornece informações valiosas sobre o impacto económico das DCV na região, utilizando um proxy da abordagem do capital humano para cálculos monetários de perda de produtividade e revelou algumas conclusões significativas.

Em 2019, a América do Sul registrou 754.324 mortes atribuídas a DCV, o que resultou em um AVPP associado totalizando 2.040.973. A perda permanente total de produtividade foi estimada em aproximadamente 3,7 mil milhões de dólares em paridade de poder de compra. Esse valor equivale a 0,11% do PIB. Estes números destacam a enorme carga económica que as DCV impõem à região. Portanto, são necessárias intervenções e políticas específicas para resolver esta questão.

A importância de medir com precisão a carga das doenças não pode ser exagerada. Vale a pena notar os desafios e limitações associados à estimativa da carga da doença. A variabilidade na qualidade e disponibilidade dos dados entre países pode afetar a precisão e a comparabilidade das estimativas. Apesar desses desafios, o estudo de Bandeira et al.,^6^ demonstra a robustez das suas conclusões em diferentes cenários, proporcionando confiança na validade dos resultados. Isto sublinha a importância de investir na colheita de dados e na infraestrutura de análise para melhorar a precisão e a fiabilidade das estimativas da carga das doenças.

Concluindo, o estudo de Bandeira et al.,^6^ destaca a necessidade urgente de ação para abordar o impacto econômico das DCV na América do Sul. Ao medir com precisão a carga da doença e compreender as suas consequências económicas, os decisores políticos podem desenvolver estratégias específicas para reduzir o impacto das DCV nos indivíduos, nas comunidades e nos sistemas de saúde.^7^ É essencial priorizar a prevenção, a detecção precoce e o tratamento das DCV para melhorar os resultados de saúde e reduzir o peso económico destas doenças na América do Sul.

A atividade física regular pode ajudar a reduzir os custos com saúde e melhorar a produtividade dos trabalhadores. Ao prevenir DCV por meio de mudanças no estilo de vida e gerir condições médicas, podemos desfrutar de uma vida melhor e de ganhos econômicos. Os empregadores também podem contribuir proporcionando um ambiente de trabalho saudável para o coração. A prevenção de doenças cardíacas pode resultar em ganhos significativos no PIB e no aumento da produtividade.

A redução das disparidades sociais através do investimento pode promover significativamente a saúde cardíaca, prevenir o sofrimento familiar, limitar a perda de produtividade e reduzir a morbidade. As desigualdades são os principais determinantes da perda de saúde. A promoção da igualdade terá um impacto positivo na saúde. Melhores resultados econônimos e educacionais para as famílias melhoram a saúde. Os problemas de saúde crônicos e as doenças não transmissíveis reduzem o rendimento dos grupos familiares, e o baixo nível socioeconômico leva a estas condições.^8 , 9^
