# Supplementary material for: O Impacto Das Doenças Cardiovasculares Nas Perdas Econômicas
Source: Arq Bras Cardiol. 2024 May 7;121(3):e20240175. [Article in Portuguese] doi: 10.36660/abc.20240175 (PMC11081202; doi:10.36660/abc.20240175)
Supplement: Translation

# Translation: The Impact of Cardiovascular Disease on Economic Loss

**DOI:** 10.36660/abc.20240175i

**Authors:** Fátima Marinho

**Affiliations:** 1 Nova Iorque USA Vital Strategies Brazil, Nova Iorque – USA; 2 Belo Horizonte MG Brazil Universidade Federal de Minas Gerais (UFMG), Belo Horizonte, MG – Brazil

**Keywords:** Cardiovascular Diseases/mortality, Public Health Policy/economics, Cost Efficiency Analysis

Cardiovascular diseases (CVD) are a public health concern that impacts the population’s health and economy. It is the leading cause of death globally.^1^In Brazil, CVD has been the leading cause of death, with Ischemic Heart Disease and Stroke as the most common causes, except during the pandemic years (2020-2021) when COVID-19 was the first cause of death for men and women.^2^

What is the impact on productive work and the economy? How can cardiovascular disease and its loss of health in the population be prevented?

CVD significantly impacts work productivity, resulting in $147 billion in lost productivity in the US annually.^3^Individuals with high cardiovascular risk experience more work loss hours and higher indirect costs compared to those without cardiovascular events. In Australia, research on preventing coronary heart disease for ten years revealed that it would save many lives and nearly USD 15 billion in gross domestic product (GDP).^4^In the European Union,^5^CVD has also had a significant impact, with estimated costs on productivity losses making up €48 billion (17%).

In South America, where the impact of CVD is particularly pronounced, efforts to quantify the economic consequences of these diseases have been limited. The study by Bandeira et al.^6^provides a valuable contribution to addressing this gap by estimating the years of productive life lost (YPLL) and associated economic costs of premature mortality from CVD in 2019.

It is important to note that there are some limitations to using data related to cardiovascular disease (CVD), which is crucial for public health policies. Not all countries have reliable data on cardiovascular disease mortality. Among 156 countries, only 70% of deaths were registered, and out of these, only 52% had medically certified causes.^7^This makes it necessary to rely on estimates of causes of death.

The research conducted by Bandeira et al.^6^utilizes data from the Global Burden of Disease Study 2019, which is a widely used and comprehensive database. The study aims to estimate the disease burden of cardiovascular disease (CVD) in South America. It provides valuable insights into the economic impact of CVD in the region by utilizing a proxy of the human capital approach for monetary calculations of productivity loss and has uncovered some significant findings.

In 2019, South America saw 754,324 deaths attributed to CVD, which resulted in an associated YPLL totaling 2,040,973. The total permanent productivity loss was estimated at approximately US$3.7 billion in purchasing power parity. This amount is equivalent to 0.11% of the GDP. These figures highlight the enormous economic burden that CVD imposes on the region. Therefore, targeted interventions and policies are necessary to address this issue.

The importance of accurately measuring the burden of disease cannot be overstated. It is worth noting the challenges and limitations associated with estimating the burden of disease. Variability in data quality and availability across countries can affect the accuracy and comparability of estimates. Despite these challenges, the study by Bandeira et al.^6^demonstrates the robustness of its findings across different scenarios, providing confidence in the validity of the results. This underscores the importance of investing in data collection and analysis infrastructure to improve the accuracy and reliability of the burden of disease estimates.

In conclusion, Bandeira et al.^6^study highlights the urgent need for action to address the economic impact of CVD in South America. By accurately measuring the burden of disease and understanding its economic consequences, policymakers can develop targeted strategies to reduce the impact of CVD on individuals, communities, and healthcare systems.^7^It is essential to prioritize prevention, early detection, and treatment of CVD to improve health outcomes and reduce the economic burden of these diseases in South America.

Regular physical activity can help reduce healthcare costs and improve worker productivity. By preventing CVD through lifestyle changes and managing medical conditions, we can enjoy a better life and annual savings. Employers can also contribute by providing a heart-healthy working environment. Preventing heart disease can result in significant gains in GDP and increased productivity.

Reducing social disparities through investment can significantly promote heart health, prevent family suffering, limit productivity loss, and reduce morbidity. Inequalities are the primary determinants of health loss. Promoting equality will have a positive impact on health. Better economic and educational outcomes for households enhance health. Chronic ill health and non-communicable diseases reduce the income status of households, and low socioeconomic status leads to these conditions.^8 , 9^
